# Destroying the androgen receptor (AR)-potential strategy to treat advanced prostate cancer

**DOI:** 10.18632/oncoscience.389

**Published:** 2017-12-28

**Authors:** Ramesh Narayanan, Suriyan Ponnusamy, Duane D. Miller

**Affiliations:** University of Tennessee Health Science Center, Memphis, TN, USA

**Keywords:** androgen receptor, selective androgen receptor degraders (SARDs), castration-resistant prostate cancer (CRPC), ubiquitin proteasome pathway

Recently, a library of selective androgen receptor degraders (SARDs) that degrade the full length and splice variant ARs (AR-SVs) through proteasome pathway was discovered and characterized by our laboratory [1]. These molecules bind, antagonize, and degrade AR at comparable sub-micromolar doses, making them potent small molecule degraders. The SARDs represent a scaffold that might result in an AR conformation that promotes degradation through ubiquitin proteasome pathway.

Understanding that despite the typical initial efficacy of androgen-deprivation therapy that the AR continues to play an important role in advance castration-resistant prostate cancer (CRPC) led to development of the next- generation AR competitive antagonist enzalutamide and the CYP17A1 inhibitor abiraterone [2]. This renewed interest in targeting the AR axis has significantly increased the efforts to discover AR antagonists. Men treated with enzalutamide and abiraterone yet are either non-responsive or develop resistance are heterogeneous in nature. Some of the observed phenotypes include a neuroendocrine type in which AR is not thought to be a driver, AR-SV-containing disease, a glucocorticoid receptor (GR)-positive subset, and others [3-4]. This heterogeneity of CRPC renders a challenge to design successful clinical trials and to develop next-generation AR-targeted drugs.

Next-generation AR-targeting molecules that are in clinical development include ARN-509 (apalutamide) [5] and ODM-201 (darolutamide). These next-generation molecules are competitive AR antagonists with no additional mechanistic attributes that would otherwise distinguish them from enzalutamide, bicalutamide, and hydroxyflutamide. Therapeutic approaches with novel distinct mechanisms of action are required to treat the rapidly changing CRPC landscape and to slow the aggressively growing forms that significantly shorten overall survival. Molecules that are mechanistically distinct from competitive antagonists have the potential to have a significant therapeutic benefit.

Although degraders to other nuclear receptors are available [6], AR degraders have been obscure and been difficult to discover and develop. Attempts to discover AR degraders have garnered interest in recent years for some of the potential mechanistic reasons depicted in Figure 1. The PROTAC technology (Arvinas) is a hybrid molecule, where a known ligand to a binding pocket is fused to an E3 ligase- recruiting molecule. This chimera will bind to the ligand binding pocket of the target protein and will recruit E3 ligase to the protein ligand complex, resulting in protein degradation [7]. Degronomids (C4 therapeutics) and SNIPERS (Takeda) were designed utilizing a similar approach [8]. These chimeric molecules are potent with nanomolar to picomolar effective doses. An apparent disadvantage of these molecules is that their molecular weight is greater than 1000 Da, which according to Lipinski's rule of five is an undesirable drug-like property for small molecules.

**Figure 1:**
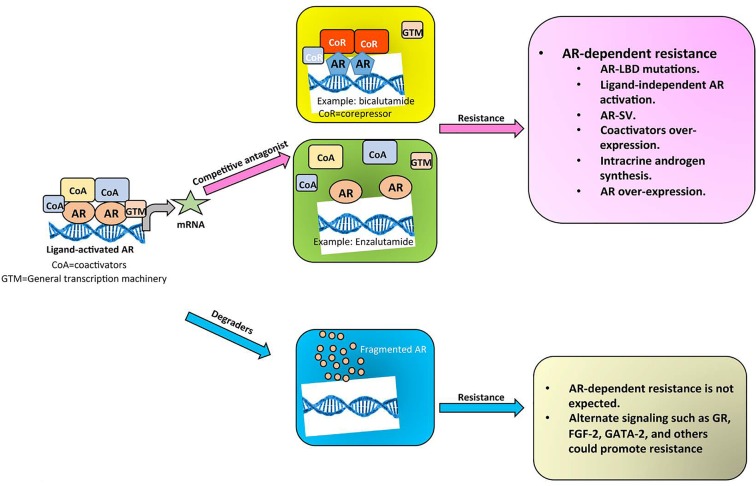
Mechanistic distinction between competitive antagonists and degraders of the AR AR bound by agonists is recruited to androgen response elements. This results in the recruitment of coactivators and general transcription machinery and increase in the transcription and translation of target genes. Competitive antagonists-bound AR either result in the recruitment of corepressors (bicalutamide) or prevents nuclear localization of AR (enzalutamide). SARDs will degrade the AR, preventing any potential resistance.

Lack of an antagonist-bound AR-LBD crystal structure makes it challenging to use structure-based drug design to develop AR antagonists or degraders. Utilizing a strategy similar to ER degraders with long side-chains to recruit ubiquitin failed to provide active AR degraders in our SARD program. Structural modifications of bicalutamide, an aryl propionamide with S-bridge, rendered modest AR degradation. However, a structure-activity relationship could not be deduced with the modified bicalutamide scaffold. Modification of the bridging atom in the arylpropionamide scaffold resulted in the first-generation molecules, UT-155 and UT-69 [1]. The molecules have N-bridge, which have been found to be important for the degradation property of the SARDs.

A surprising feature of UT-69 and UT-155 is their ability to degrade the AR-SVs. In CRPC patients, the AR-SVs have been observed only in the presence of full length AR. This observation led to hypothesis that the AR-SV degradation with the SARDs could have been due to destabilization of the heterodimer as a result of AR degradation. However, results from engineered cells expressing only an AR-SV suggest that these molecules might have a second binding site. We narrowed this binding site down to the activation function-1 (AF-1) region in the N-terminal domain (NTD). Again, lack of a crystal structure of the NTD, an intrinsically disordered domain, makes it difficult to visualize the conformational changes in the presence of such binders. It is unclear whether the NTD possesses a binding pocket that can be utilized to design molecules or that the SARDs have a non-selective affinity to certain amino acid sequence in the NTD. Despite the inability to demonstrate a direct binding of SARDs to the AF-1 region due to the lack of a competitive-binding assay, interaction of the SARDs with the AF-1 was shown using three independent biophysical methods. The SARDs promoted degradation of the AR and AR-SV under multiple conditions, including under conditions of activated kinases and mutated AR-LBD [1]. The domain that contributed to the antagonistic action of the SARDs differed between scaffolds with AF-1- dependent degradation being important for UT-155, while competitive antagonism if sufficient for the function of UT-69.

Therapeutic potential of molecules that potently degrade the AR can be extended beyond CRPC to other maladies, such as a subset of triple-negative breast cancer, Kennedy's disease, male-pattern baldness, and others. The degraders could also be used as the first-line therapy in early androgen-responsive prostate cancer, where gonadotrophins are the treatment of choice. Based upon structure alone, SARDs belonging to the UT-69 and UT- 155 scaffold may not elicit CNS side-effects, we believe that the toxicity is due to the hydantoin backbone of enzalutamide and apalutamide and due to 7-aminobutyric acid receptor cross-reactivity. Other side-effects such as muscle weakening and osteoporosis might be encountered and will be discerned as studies progress. These side- effects could be overcome with potential disease-directed therapies. A SARD could provide an effective therapy for CRPC patients and could become the treatment of choice in future.

## References

[1] Ponnusamy S (2017). Cancer Res.

[2] Tran C (2009). Science.

[3] Bluemn EG (2017). Cancer Cell.

[4] Vidal SJ (2015). Cancer Cell.

[5] Balbas MD (2013). Elife.

[6] Bihani T (2017). Clin Cancer Res.

[7] Raina K (2016). Proc Natl Acad Sci U S A.

[8] Shibata N (2017). J Med Chem.

